# Editorial: Women in neuroscience of Bioelectronic Medicine

**DOI:** 10.3389/fnint.2024.1406013

**Published:** 2024-04-16

**Authors:** Stéphanie C. Thébault, Marie-Ève Tremblay, Silvia V. Conde, María Alejandra González-González

**Affiliations:** ^1^Laboratorio de Investigación Traslacional en Salud Visual (D-13), Instituto de Neurobiología, Universidad Nacional Autónoma de México (UNAM), Querétaro, Mexico; ^2^Division of Medical Sciences, University of Victoria, Victoria, BC, Canada; ^3^Department of Neurology and Neurosurgery, McGill University, Montreal, QC, Canada; ^4^Department of Molecular Medicine, Université Laval, Québec City, QC, Canada; ^5^Department of Biochemistry and Molecular Biology, The University of British Columbia, Vancouver, BC, Canada; ^6^iNOVA4Health, NOVA Medical School, Faculdade de Ciências Médicas, NOVA University, Lisbon, Portugal; ^7^Jan and Dan Duncan Neurological Research Institute, Texas Children's Hospital, Houston, TX, United States; ^8^Department of Pediatric Neurology, Baylor College of Medicine, Houston, TX, United States

**Keywords:** Bioelectronic Medicine, neuromodulation, neurophysiology, women in science, biomedical treatments, electroceuticals

Bioelectronic Medicine is becoming the landmark of a new era of therapies, where pharmacological treatments are replaced or combined with the leverage of electricity to restore optimal function of organs and systems. The list of applications is becoming countless, converging with the necessity of mechanistic biological understanding, the advancement of technologies in biocompatible and conductive materials, the integration of computational and electronical approaches, and the translation of basic science into the clinic. The complexity behind the development of translational treatments relies on its inherent multidisciplinary nature, which has prompted international instances to foster the creation of centers and programs to enhance collaborative efforts. The Research Topic: “*Women in neuroscience of Bioelectronic Medicine*” was initiated not only to promote the integration of neurosciences with multiple other disciplines, but also to encourage the participation of women, historically underrepresented in science. This Research Topic has 100% participation of women in the editorial, 80% as first author, and 60% of all authors.

The nervous system conveys exquisite specialization to integrate multiple functions and maintain systemic homeostasis, and our Research Topic features research in both central and peripheral nervous systems. Neuroscience is often associated with the study of the brain. However, the study of peripheral (somatic and autonomic) nerve circuitry and brain-peripheral interactions is equally important to understand systemic disorders and develop precision therapies with little to no off-target side effects. The research presented by Rodriguez-Arzate et al. is an example, they discuss the use of non-photic electroretinogram (ERG) as a non-invasive method commonly used in eye care, for the high-resolution capture of retinal function. Their work compared *spontaneous retinal oscillations* from different species and proposed this approach as a biomarker to study dysfunction in retinal pathophysiology and to diagnose retinal and other neuronal pathologies at early stage (i.e., hereditary retinal dystrophy and multiple sclerosis). Seicol et al. present a perspective on the use of neuromodulation to reduce inflammation and prevent hearing loss due to inflammation in the auditory system. On the peripheral nerve side, Alvarado-Navarrete et al. integrate concepts on the spinal cord and vestibular input through the study of Hoffman's reflex to evaluate transient excitability outputs on descending pathways. This allowed them to identify biomarkers in the evaluation of the integrity of vestibulospinal tracts, often disrupted in injured patients or during neurodegenerative diseases.

Our Research Topic also features a special and comprehensive multidisciplinary review by González-González et al. “*Bioelectronic Medicine: a multidisciplinary roadmap from biophysics to precision therapies*.” This review brings together scientists from different fields to discuss concepts with relevance to Bioelectronic Medicine. The highlights include a historic literature compilation of this emerging field, followed by core concepts on the biophysics of the neuronal membrane presented by the esteemed biophysicist Ramon Latorre. A special emphasis is provided to the *refractory period*, referring to the inactivation of neurons immediately after all voltage dependent Na^+^ channels are opened due to an action potential event. This concept is very relevant to the design of neuromodulation treatments, often modifying frequency, amplitude, and pulse duration of the electrical stimulus.

The phenomenon of *neurotransmitter switching* was discovered by the group of Nicholas Spitzer, who, along with Marta Pratelli, discussed in the review the importance of taking this phenomenon into account when designing long-term treatments. Their research opens the possibility to understand nervous system plasticity through the lens of neuromodulation that modifies the nature of neurotransmitters and their corresponding receptors in the post synapse. This concept could impact the design of psychiatric treatments, and hopefully replace or reduce the use of neuroactive drugs with deleterious side effects or limited efficacy. González-González et al. further provide an overview of core concepts pertaining to the physiology of the cell and the advancement in system neuroscience. They discuss the concept of *field potentials*, which is the temporal summation of synchronous activity among neuronal populations modulated not only by the activity of glial and endothelial cells, but also by circulating molecules in the blood flow capable of crossing blood barriers and transferred to neurons by specialized neuroglia. The role of *neuroglia* is presented by González-González et al., pioneers, and lifelong experts in the study of these specialized non-neuronal cells in the nervous system. They provide an overview of the concepts, classification, and functions of the various types of neuroglia, including microglia, which are highly relevant to various pathologies. The modulation of microglial structure and function by an emerging neuromodulation modality, transcranial focused ultrasound stimulation, is further discussed by Grewal et al.. In their review, the authors cover the outcomes of this therapeutic strategy targeting specific regions of the brain in a minimally invasive manner on various microglial properties, including their surveillance of the brain and phagocytosis, across contexts of health and disease.

The large amount of complex data collection, its analysis and integration with bioelectronic devices is discussed by González-González et al., a computer scientist expert in data science of complex networks and large-scale graph analysis. He presents the concept of *Internet of Things* (IoT), referring to the integration of interconnected devices capable of communicating information to other communication networks, serving as a powerful resource merging with the field of Bioelectronic Medicine to monitor and design real-time personalized treatments. The transition of therapies to the clinic, linked to the development of optimized biocompatible materials (by González-González et al.) and the validation of electrochemical properties of conductive materials for safe electrode-tissue interface unit (by González-González et al.) are also discussed. Regulatory processes for medical devices and preclinical studies are lastly discussed by González-González et al., respectively, finalizing with point-of-care by the clinical neurosurgeon Ma. This comprehensive review builds upon multiple pieces relevant to the design and implementation of a bioelectronic treatment and is projected as a guide for both experts and less experienced young scientists. We envision this Research Topic to promote the multidisciplinary work and the increase of women participation in the Bioelectronic Medicine field.

## Author contributions

ST: Writing – review & editing. M-ET: Writing – review & editing. SC: Writing – review & editing. MG-G: Conceptualization, Writing – original draft, Writing – review & editing.

